# ‘Doing dying well’: Positive narratives of specialist palliative care occupational therapy from Ireland-based practitioners

**DOI:** 10.1177/03080226251412164

**Published:** 2026-01-29

**Authors:** Eoin Gorman, Clodagh Daly, Ciara Walsh

**Affiliations:** 1 University College Cork, Cork, Ireland; 2 Beaumount Hospital, Dublin, Ireland; 3 University Hospital Limerick, Limerick, Ireland

**Keywords:** Palliative care, occupational science, occupational therapy, occupational engagement, dying, client-centred

## Abstract

**Introduction:**

Occupational therapists working in specialist palliative care aim to enable clients with life-limiting illnesses to continue engaging in meaningful occupations. However, there is a dearth of literature illustrating positive examples of occupational therapy practice in specialist palliative care in Ireland. This study aimed to highlight the positive examples of occupational therapists assisting clients in ‘doing dying well’ in specialist palliative care.

**Method:**

Single semi-structured interviews were conducted with nine occupational therapists, working in six different specialist palliative care settings. Interviews were transcribed verbatim and analysed using thematic analysis.

**Findings:**

The first theme identified occupational therapists’ perceptions of how clients continue to ‘*focus on living*’ when dying. The second theme identified the core elements of ‘*being client-centred*’ as (i) ‘being present’, (ii) ‘developing collaborative goals’ and (iii) ‘supporting families and caregivers’. The third theme ‘*holism*’ described clients’ (i) ‘holistic everyday occupations’ and (ii) ‘holistic unique occupations’ in ‘doing dying well’.

**Conclusion:**

Occupational therapists perceived that ‘doing dying well’ for clients can be facilitated by focusing on living until death, enabling continued engagement in occupations and roles. Adopting a truly client-centred and occupation-focused approach, occupational therapists can enable dying individuals to live and die as they want.

## Introduction

Individuals approaching death face continual challenges to their physical, psychosocial and spiritual self (World Health Organization, 2020). Palliative care enhances quality of life for people with life-limiting illnesses and their families; achieved by means of preventing and alleviating suffering and addressing spiritual, physical and psychosocial needs (Health Service Executive, 2018), with specialist palliative catering for people with complex needs who are dying because of a life-limiting illness (Irish Hospice Foundation, 2014). Specialist palliative care occupational therapists support continued engagement in desired occupations for people who are dying and their families.

Definitions of dying include descriptors such as ‘approaching death’ or ‘ultimate stage of decay or disuse’ describe dying as a ‘non-occupational or passive process’ (Pollard, 2006) lacking choice and agency. Casey et al. (2011) framed ‘dying well’ as holistic, individualised care considering comfort, culture, dignity and environment. Irish initiatives in specialist palliative care champion the importance of ‘dying well’ (Damery et al., 2023) with The ‘Hospice Friendly Hospitals’ and ‘Dying Well at Home’ programmes aiming to provide spaces designed to ensure dignity and respect and support a rewarding and comforting experience, respectively, for dying individuals and their families and carers. Additionally, a report from the Office of the Ombudsman reflected public complaints relating to end-of-life care in Irish hospitals highlighting the long-lasting emotional effects of negative end of life. While progress has been made in healthcare arenas in terms of communication and organisational structures (Office of the Ombudsman, 2018), scope exists to enhance the welfare and dignity of dying individuals, hence, the need to explore and highlight positive examples of experiences during death.

To capture the core elements of ‘dying well’ and to foster an occupational and client-centred perspective, in this study ‘dying well’ is reframed as ‘doing dying well’ (Hitch et al., 2014). Re-framing as ‘doing dying well’ broadens the concept to include meaning, context, human relationships, choice and agency, moving the focus from a limited, passive and physical process to a unified, inclusive concept. Existing literature in specialist palliative care typically focuses on theory relating to death and dying, along with practical elements such as barriers and facilitators in the provision of occupational therapy. Research suggests that as a client approaches death, priorities change from focusing on life to preparing for death but the desire for continued occupational engagement may intensify as death approaches (Hammill et al., 2019; La Cour et al., 2009; Pollard, 2006) supporting the view that humans are inherently occupational, regardless of stage of life. Continued occupational engagement can provide individuals facing death with a sense of purpose (Von Post and Wagman, 2019), and the imminence of death can change the focus and meaning of everyday occupations (La Cour et al., 2009; Mills and Payne, 2015; Park Lala and Kinsella, 2011; Pollard, 2006). Indeed, dying individuals have been noted to experience an increased focus on the quality of occupations and a new appreciation for the seemingly ordinary occupations of everyday life (Mills and Payne, 2015; Park Lala and Kinsella, 2011; Pollard, 2006), and has been observed in how everyday occupations were used to express relationships, address spiritual needs, obtain a sense of closure and express an intense experience of ‘being’ (Pizzi, 2014; Pollard, 2006).

‘Occupational disruption’ refers to a situation in which a person is unable to participate in meaningful occupations, fully or partially, due to injury or illness (Nizzero et al., 2017). A life-limiting illness is a ‘catastrophic change’ significantly altering one’s occupational situation and contributes to significant occupational disruption in the imminence of death, predominantly from the loss of physical ability (Costa and Othero, 2012; Keesing and Rosenwax, 2011; La Cour et al., 2009). Occupational disruption negatively impacts the dying person’s daily routine and engagement in valued occupations and disengagement or withdrawal from friendships, intimate relationships and family life, resulting in changes to the person’s identity (Keesing and Rosenwax, 2011). ‘Occupational adaptation’ is a response to occupational disruption for individuals facing death, resulting in continued occupational engagement (Grajo et al., 2018). Dying individuals adapt or develop new ways of performing meaningful occupations and revise expectations of their performance, and have been noted to counter reduced physical capacity by prioritising and adjusting activities, along with modifying their environment (Hammill et al., 2019). A client-centred approach is used to foster collaboration between the client and therapist, facilitating continued occupational engagement (Mills and Payne, 2015; Pizzi, 2015; Treggalles and Lowrie, 2018). Occupational therapy interventions included assistive equipment and environmental modifications (Eva and Morgan, 2018; Pizzi, 2015; Tavemark et al., 2019) and symptom management (Eva and Morgan, 2018). Occupational therapists also provide education and support to caregivers and families, to promote clients continued occupational engagement (Eva and Morgan, 2018; Hammill et al., 2019; Pizzi, 2015) as well as supporting families during the client’s illness and following the death of the client (Eva and Morgan, 2018; Hammill et al., 2019; Pizzi, 2015).

Conversely, literature suggests occupation is not always at the centre of occupational therapy practice in palliative care (Badger et al., 2016). Barriers within specialist palliative care occupational therapy were attributed to personal, team and systemic factors. Occupational therapists may avoid discussions relating to death and dying as a method of self-protection and to manage emotional vulnerability and distress regarding personal fears surrounding death (Casey et al., 2011; Treggalles and Lowrie, 2018). Consequently, clients may feel uncomfortable discussing their concerns or goals due to a lack of trust and rapport with the occupational therapist (Treggalles and Lowrie, 2018). Ambiguity and obscurity surrounding the scope of occupational therapists’ work within specialist palliative care settings diminish the unique significance and contribution of occupation for clients facing death (Keesing and Rosenwax, 2011; Pizzi, 2014; Talbot-Coulombe et al., 2022), with understaffing, a lack of material and human resources, large caseloads and poor funding further restricting occupational therapists’ scope of practice and the number of contact hours spent with clients (Casey et al., 2011; Eva and Morgan, 2018; Talbot-Coulombe et al., 2022). Such constraints may result in interventions which are less focused on occupation and meaningful occupational engagement (Casey et al., 2011; Eva and Morgan, 2018).

The focus of specialist palliative care occupational therapy on engagement in meaningful occupations for the clients positions it uniquely to explore how these therapies potentially benefit dying individuals and improve the dying experience for both them and their families. Studies to date typically focus on theoretical and practical issues relating to the provision of occupational therapy and death and dying, with limited international research exploring practitioner experiences of providing occupational therapy in specialist palliative care (Keesing and Rosenwax, 2011; Martin and Herkt, 2018). Furthermore, there is limited research highlighting positive examples of providing occupational therapy within specialist palliative care. These examples may contribute to healthcare advancements beyond the realms of occupational therapy, providing space and reflection for both clients, practitioners and educators to explore the role of occupations in refining and personalising the death experience in a client-centred approach. Therefore, the primary aims of this study were to explore occupational therapists’ understanding and practice of ‘doing dying well’ with clients in specialist palliative care and to illuminate positive examples of such practices.

## Method

### Research design

A qualitative descriptive approach, underpinned by a constructivist paradigm, was chosen to capture the voices of those experiencing the phenomena of interest, with semi-structured interviews used to discover participants’ reflections, thoughts and feelings (Holloway and Galvin, 2017) on positive examples of occupational therapists facilitating clients in ‘doing dying well’. The research team consisted of a male university lecturer and two final-year, female undergraduate occupational therapy students trained in qualitative research methodologies through their academic programme. The students conducted the interviews under supervision of the lecturer, who oversaw the study design, ethical approval and analysis. Interviews were conducted over a 3-month period from February to April 2019. The period between data collection and manuscript submission in 2024 involved intermittent but ongoing analysis, with progress affected by unforeseen professional constraints and the impact of the COVID-19 pandemic. Despite the time elapsed, data remain relevant as the stability of the phenomena are not subject to rapid temporal changes ensuring findings remain applicable, the data capture a snapshot of perspectives which may inform longitudinal comparisons, and the research questions and findings remain central to contemporary scholarship. This study adhered to the Consolidated Criteria for Reporting Qualitative Research (Tong et al., 2007) and the Standards for Reporting Qualitative Research (O’Brien et al., 2014) guidelines to ensure transparency, rigor and completeness in the reporting of qualitative research.

### Recruitment

Inclusion criteria stipulated occupational therapists working in specialist palliative care settings in Ireland for a minimum of 12 months in the last 4 years. Participant recruitment adopted two approaches: purposive and snowball sampling (Carpenter and Suto, 2008). Purposive sampling involved contacting members of the Association of Occupational Therapists Ireland Palliative Care Advisory Group, via email, through a gatekeeper. The email included a study information letter. Snowball sampling was also employed due to the limited number of potential participants who met the inclusion criteria for the study within the Irish context. Potential participants contacted the research team directly or provided permission to be contacted via a relevant third party, with 13 participants expressing interest to participate in the study. Detailed information on the study was distributed, and the first 10 occupational therapists who provided informed consent were formally recruited. Those who expressed interest after the first 10 were thanked and informed that the recruitment target had been met. All participants were women employed across a range of specialist palliative care units, hospices and community settings throughout Ireland with experience in this setting ranging from 3 to 21 years. Pseudonyms were used to represent individual contributions. Given the small pool of occupational therapy specialist palliative care workforce in Ireland, further demographic details (including geographical location) are limited or described collectively to ensure confidentiality. One participant withdrew from the study during the data collection timeline, and on review of preliminary data, it was decided by the research team that data saturation was achieved using nine participant interviews. Saturation was assessed through systematic monitoring of recurring patterns, completeness of thematic coverage and adequate representation of diverse participant perspectives.

### Data collection

**Figure 1.**
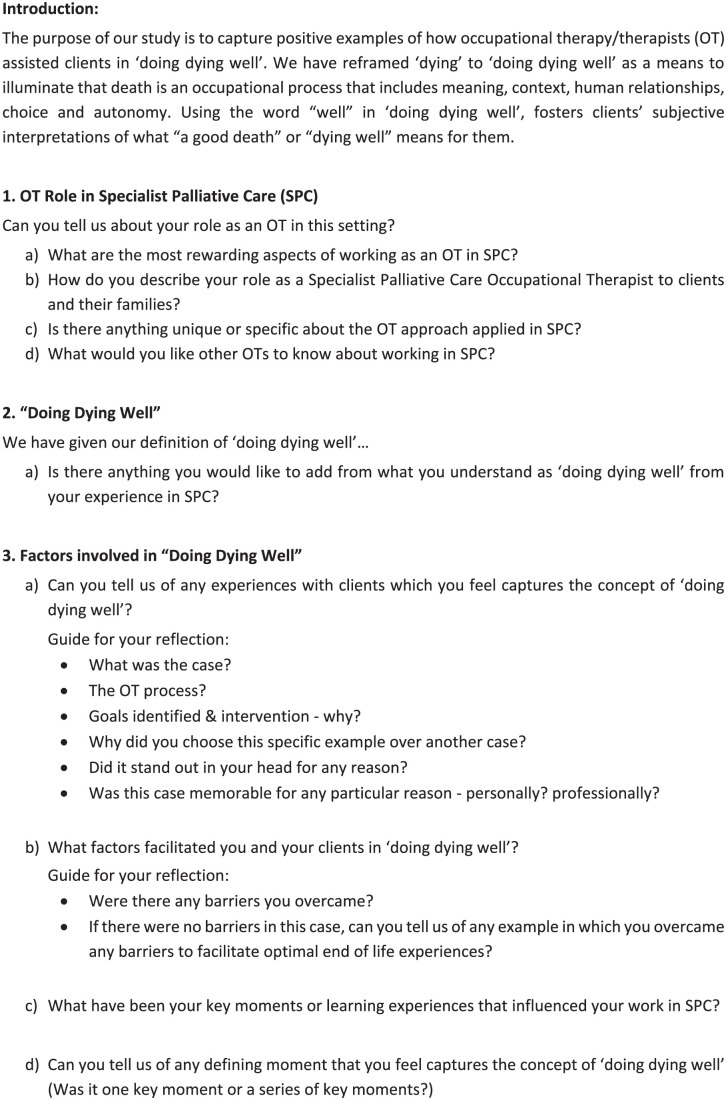
Interview guide for semi-structured interviews conducted with occupational therapists working in specialist palliative care.

Semi-structured interview questions were piloted with an occupational therapist with experience in specialist palliative care. Questions were amended and refined based on feedback, prior to data collection and pilot interview data was excluded from subsequent analysis. An interview guide (Figure 1) was sent to participants in advance of each interview to allow time for reflection. Participants were offered the option of face-to-face or online interviews, to allow for flexibility. Data collection consisted of eight face-to-face interviews and one online interview. Each interview had a duration of approx. 40 minutes, was audio-recorded and transcribed verbatim, with participants’ consent. No prior relationships existed between the interviewers and participants. Participants were informed of the interviewers’ professional background and research interests in the study information letter. All interviews were conducted in agreed private settings with no non-participants present during the interviews. Each participant was interviewed once only. Field notes were taken during and immediately after each interview to capture contextual details and initial impressions to support data analysis. Transcripts were not returned to participants to preserve the temporal authenticity of their reflections, without retrospective alteration. Consequently, participants did not participate in the validation of the final themes. All data were securely stored in accordance with the university’s ethical guidelines.

### Data analysis

Thematic analysis was used to identify, analyse and interpret patterns within the interview data. While the study initially followed Braun and Clarke’s (2006) framework, the research team also engaged with recent developments in reflexive thematic analysis (Braun and Clarke, 2019, 2021) to enhance analytical rigour and transparency. Data analysis was undertaken manually, with printed transcripts systematically hand-coded by student researchers under remote supervision, employing an inductive and interpretive analytical framework. Similar codes were grouped through reading and annotation, to create categories of interconnecting data and then amalgamated, identifying themes and subthemes (Stanley and Nayar, 2014). During data analysis, all researchers considered both the latent and manifest aspects of the data, which enabled participants’ views on the topic to be identified through layers of interpretation and exploration (Braun and Clarke, 2006; Stanley and Nayar, 2014). Following Braun and Clarke’s (2019, 2021) updated guidelines, recognising themes as conceptualised as patterns of shared meaning, the identified themes were reviewed through iterative, reflexive engagement and collaborative discussion amongst the research team. Alternative perspectives, explanations and biases were explored prior to constructing the final themes. The team’s lecturing expertise and education in the topic of death and dying were acknowledged as potential sources of bias, however, reflexive practices were employed to mitigate these influences and to ensure that participants’ voices were represented authentically.

### Ethics

Ethical approval was granted from the university’s Social Research Ethics Committee (approval number: CT-SREC-2018-19). Participants were assured of anonymity, and voluntary participation was emphasised through the study information. Informed consent was obtained from all participants. Any potentially identifying information was removed or anonymised during analysis and reporting.

## Findings

Three themes with corresponding subthemes were constructed during analysis (Table 1). Pseudonyms are used to convey the voices of participants and ensure anonymity.

**Table 1.** Themes and subthemes constructed during analysis of semi-structured interviews conducted with occupational therapists working in specialist palliative care.

Themes	Subthemes
Focus on living	■ Continuing occupational engagement ■ Change in meaning of occupations
Being client-centred	■ Being present ■ Developing collaborative goals ■ Supporting families and caregivers
Holism	■ Holistic everyday occupations ■ Holistic unique occupations

### Focus on living

While specialist palliative care is associated with death and dying, participants considered the nature of their work as occupational therapists is to focus on living. This is achieved by enabling continued occupational engagement until death. Occupations are at the centre of doing dying well or doing living well as Sarah expressed, ‘*We try to focus on living . . . so it’s doing dying well but also doing living well until the end of your life*’. Rather than ‘doing dying well’ with clients, participants focus on ‘doing living well’ through occupational engagement. Participants centred occupation and attributed clients’ desire to continue occupational engagement as a means of maintaining ‘normality’ in the face of death. One participant described how, for one client, engaging in everyday ‘normal’ occupations with her children enabled her to live until the end of her life:

She wanted to keep it more normal for the kids. . . she went to the shop to buy them new winter jackets, you know those kinds of things. They were just the normal, everyday things she would do as a mum. She was living to the very end and keeping her role as a mum. (Maria)

Another example was provided detailing how a client maintained his role as a father by adapting to his reduced functional ability to continue engaging in meaningful occupations:

They are still a father, they’re still trying to do all them things from here and they might not be as able but seeing how they can integrate that, and even they can maybe do half of it or do some of it, or maybe teach their wife how to do it, while still maintaining that role. (Alice)

Participants considered that in adapting and continuing engagement in meaningful occupations, clients strived to maintain their roles and identities in the face of death. Participants noted their clients’ wish to focus on living was intertwined with a change in the meaning of occupations. This change in meaning included an increased appreciation for continuing engagement in seemingly mundane, everyday occupations, described by participants as ‘the little things’. Emma provided examples of ‘the little things’ in doing dying well, ‘*so it’s a walk in the garden, it’s sitting out in a chair, it’s having a birthday party . . . that’s doing dying well or doing living well*’. Participants commented on clients attaining a sense of achievement from doing ‘little things’. Chloe expressed, ‘*everyday things become hugely important because that’s your achievement. Getting dressed, getting washed, getting out of bed*’.

Emma noted the change in meaning in ‘the little things’ may be attributed to a deterioration in a client’s functional ability as their illness progresses and death becomes more imminent, ‘*I think it shifts really to where people are at and especially if they’re unwell, sometimes their bucket list just becomes the real little things*’. All participants recognised their clients’ appreciation for seemingly ordinary, everyday occupations appeared to be enhanced in the imminence of death. Participants were attentive to the new significance placed on ‘the little things’ with regards to clients’ everyday occupations in ‘doing dying well’.

### Being client-centred

In attending to occupations or ‘the little things’ identified as important by clients, participants recognised ‘being client-centred’ as essential to ‘doing dying well’. Participants described being client-centred throughout the occupational therapy process, as (i) being present, (ii) developing collaborative goals and (iii) supporting families and caregivers.

Participants described ‘being present’ as spending time with clients, getting to know the person and their narrative. Participants explained that ‘being present’ provides clients with the opportunity to discuss any concerns they may have. Sarah noted, ‘*I might see half the amount of people, but I actually sit with them and hear their story and. . . just letting people say what they need to say*’. Additionally, participants identified actively listening to the client as another aspect of ‘being present’. Active listening was noted as providing greater insight into clients’ meaningful occupations. Sophie explained, ‘*It’s about hearing from an occupational therapy perspective what is unsaid*’. ‘Being present’ was also reported by participants to facilitate the development of the therapeutic relationship. Participants noted that developing a therapeutic relationship positioned clients’ wishes at the forefront, contributing to successful interventions. Alice surmised, ‘*going gently as well because developing that rapport is the most important part of your intervention, you know, because then you can actually get to where you want to b*’. Participants agreed a client-centred approach was guided by clients’ desires and wishes. Consequently, participants described ‘developing collaborative goals’ as important for respecting clients’ autonomy and personal choice throughout the occupational therapy process. Emma noted, ‘*It can be anything and everything . . . for me it’s whatever that is for that patient, if we can achieve that at some level then that’s doing dying well or doing living well*’.

Participants recognised clients may experience considerable loss and disempowerment in receiving a life-limiting diagnosis. Consequently, participants noted it is important to empower clients for clients to regain a sense of control in their life. Client-led goal setting was considered a means for the dying individual to reclaim control and feel empowered. Chloe stated, ‘*There’s so much loss of control. Offering the person choice, and offering the person, you know, options, is hugely important, because we see them at the end of the journey, and they’ve lost so much control along the way*’.

Client-centredness was understood as an important, iterative process, extending beyond initial goal setting. All participants stressed the need to continually re-evaluate and develop collaborative goals with clients as their physical and psychosocial needs change, and with the imminence of death:

I think client-centredness is a massive factor because the needs of your patients are constantly changing, so you’re constantly having to review with the patient what their goals are, what they would like to be able to do. You’re also constantly reviewing what they are actually physically able for. (Chloe)

Participants highlighted that client-centred ‘doing dying well’ extends beyond working individually with the client to include clients’ families and caregivers. Participants noted their role includes educating and supporting family members, to promote clients’ ongoing engagement in valued occupations. Lucy noted, ‘*It’s the patient’s goals but also, there’s a big body of work as well with family and their needs and education and support for the family*’. Participants also emphasised the importance of post-death family support:

I was meeting the family on a weekly basis . . . they get to know people through the disease and when the patient dies, they have a huge sense of loss because they are not meeting all these people, so it’s about working with families even after the patient has died. (Chloe)

Client-centred ‘doing dying well’ includes post-bereavement visits with bereaved clients’ family members, allowing participants to acknowledge the sense of loss experienced by the family and caregivers.

### Holism

By keeping clients’ occupational needs and wishes at the fore of the therapy process, participants noted clients directed interventions in one of two ways: (i) maintaining function in activities of daily living and (ii) doing occupations with special meaning for the client.

Participants addressed clients’ wishes to maintain function in activities of daily living, described as ‘the little things’ such as toileting, showering, dressing and fulfilling occupational roles. One participant recounted how she facilitated her client’s wishes to maintain his independence:

It was even simple little things like providing a self-propelling shower chair to go in and out of the bathroom, he was able to transfer independently, so those little things, they were so important to him. Just a bit of privacy. (Alice)

Maintaining function and occupational engagement was often addressed through complex symptom management including, ‘*fatigue management, role adjustment, breathlessness, anxiety and stress management, and complex seating*’ (Lucy). Participants noted holistic approaches to symptom management addressed clients’ physical health and psychosocial well-being, and their ability to engage in everyday occupations. Examples provided of adopting a holistic approach to enable engagement in occupations with special meaning included family events, birthday parties, christenings and weddings. Two particularly illustrative examples were provided by Sarah and Lucy.

Sarah described how being client-centred and holistic resulted in facilitating a client to achieve a lifelong goal of showcasing his art in a private exhibition. Sarah addressed the client’s physical needs in achieving the goal, ‘*It’s something he never thought he would do even when he was well and didn’t think he would be able to do considering he was dying*’, while simultaneously addressing his psychosocial needs, ‘*but it was just about giving him the opportunity to identify something really meaningful to him and support him through it*’.

Lucy’s example described enabling a client to renew her marriage vows to express commitment to her partner. Lucy continually re-evaluated the client’s seating system, upper limb strength and safety with transfers and mobility as the client’s needs changed. Sitting tolerance and fatigue management were also assessed and a wheelchair accessible taxi was arranged to go shopping for a wedding dress. Additional holistic interventions included assisting the client in writing vows and choosing music and decorations for the ceremony. The client’s family and friends attended the ceremony and engaged in celebratory activities after the ceremony.

## Discussion

Findings from this study suggest specialist palliative care occupational therapists understood ‘doing dying well’ to comprise a ‘focus on living’ in a ‘client-centred’ and ‘holistic’ manner. Embedded throughout these findings was participants recognition of the importance of continued occupational engagement for clients, highlighting occupation as the core of practice for successful therapeutic interventions.

While specialist palliative care is associated with death and dying, participants identified that clients want to ‘focus on living’, rather than dying, through continued occupational engagement. Participants proposed that ‘doing dying well’ can be reframed as and achieved by ‘doing living well’. This re-framing suggested by participants proposes that dying and living can be understood synonymously; both involving choice, agency and meaning. Similarly, Pollard (2006) suggested that dying can be re-framed as ‘living intensely’ (2006: 149). Participants noted clients wish to ‘focus on living’ through engagement in ‘the little things’ to maintain normality and identities until death. This finding suggests that ‘doing dying well’ centres on engaging in occupations that affirm life even while dying and up until death. Contrastingly, Hammill et al. (2019) noted occupational therapists perceive a shift in clients’ priorities from occupations that focus on living to preparing for death, such as writing a will or completing funeral arrangements. Participants did not perceive such a shift in focus from living to preparing for death among clients but reported that although clients were dying, the desire to focus on living did not diminish or fade in the face of death; the desire remained until death.

Findings from this study are congruent with research which suggests that dying individuals are motivated to adapt to their circumstances to continue occupational engagement (Grajo et al., 2018; Hammill et al., 2019; La Cour et al., 2009; Von Post and Wagman, 2019). Participants noted continued engagement in occupations for clients with a life-limiting illness was often impacted by reduced functional ability. Focusing on living through engagement in meaningful occupations initiated a process of facilitating occupational adaptation to overcome occupational disruption. Participants noted clients’ desire for continued occupational engagement constitutes acknowledging and exploring a change in the meaning of occupations, resulting from having to face their own mortality. Re-evaluation of the meaning and significance of ‘the little things’, for example, getting dressed or sitting in the garden, by dying individuals created expanded scope for attainment of a sense of accomplishment and positive emotions through occupational engagement. This finding is comparable to Hammill et al. (2019) in which occupational therapists noted clients placed less importance on productive tasks and chose to focus on simple occupations to regains a sense of control and focus on living. Additionally, time has been identified as an important factor for dying individuals as clients have increased emotional significance and meaning in their everyday occupations (Mills and Payne, 2015; Park Lala and Kinsella, 2011). By focusing on engagement in ‘the little things’, healthcare practitioners could further facilitate meaningful experiences for clients in ‘doing dying well’.

Adopting a client-centred and holistic approach to collaborative goal setting was found in this study to lead to bespoke interventions that are naturally focused on occupational engagement. Congruent with research by Mills and Payne (2015), participants recognised that being client-centred involved developing collaborative goals with the dying individual, surmising that allowing the client to lead the goal-setting process, empowered and enabled the client to regain significant control and choice. Interestingly, the literature suggested that occupation is not always at the centre of occupational therapy practice in palliative care (Badger et al., 2016; Carpenter, 2014; Keesing and Rosenwax, 2011; Park Lala and Kinsella, 2011); however, there are many external factors which may impede this process. Talbot-Coulombe et al. (2022) found that, in a Canadian setting, referrals may be made later in the client’s palliative care, or the general palliative care process may not be functioning optimally, leading to increased restrictions on the scope of interventions. Furthermore, health status and functional ability of dying individuals can fluctuate quickly which may further limit scope for interventions (Den Herder-Van Der Eerden et al., 2018). Ultimately, it has been noted that some healthcare professionals, and even some occupational therapists, may not fully understand the role and benefits of occupational therapy within palliative care (Knecht-Sabres et al., 2019; Mills and Payne, 2015; Talbot-Coulombe et al., 2022). While these factors may impede successful interventions, it has been noted by dying individuals that when occupational therapists fail to adopt a client-centred approach to goal setting, the clients feel disempowered in the therapy process (Badger et al., 2016). This study provides positive examples of occupational therapy practice in specialist palliative care in Ireland, facilitating ‘doing dying well’ through working collaboratively with clients, even for ‘the little things’, to keep occupation at the core of a client-centred and holistic process. This approach retains the psychosocial needs of the client at the centre of the invention, addressing needs which palliative care clients and their families have previously stated as crucial for optimum end of life care (Virdun et al., 2017, 2020).

## Limitations

Recruitment through the Association of Occupational Therapists of Ireland Palliative Care and Oncology Advisory Group may have influenced the profile of participants; however, the group includes occupational therapists working across diverse settings, including hospices, acute hospitals, community services and academic institutions, who have clinical experience or interest in palliative care. Consequently, their contributions offer valuable perspectives on clinical practice and outlooks in this specialised area. Owing to the specialised nature of occupational therapy in palliative care in Ireland, the potential pool for recruitment was limited. However, participants brought rich, practice-based insights that aligned with the study’s aims. The depth and quality of the data supported meaningful theme construction and contributed to nuanced understandings of occupational therapy practice in specialist palliative care.

## Implications for clinical practice

The core components of ‘doing dying well’ provide a potential framework to guide therapists working in palliative care promoting a ‘focus on living’ while adopting a ‘client-centred’ and ‘holistic’ approach that centres on occupational engagement. These findings have implications for specialist palliative care practice in Ireland and internationally, building upon the knowledge base of occupational therapy practice associated with death and dying. Understanding the components of ‘doing dying well’ can assist therapists to acknowledge the centrality of occupation and continued occupational engagement for clients facing death. Findings could also enable therapists to understand that the meaning of occupations will change for clients when facing death, and the importance of doing ‘the little things’ and occupations with special meaning. Furthermore, therapists may gain an understanding that ‘doing dying well’ incorporates client-centred, holistic goal setting and interventions. The ‘doing dying well’ approach taken by the participants in this study could influence client-centred interventions in other healthcare disciplines, while also promoting the important role of occupational therapy within multi-disciplinary teams in palliative care.

It is recommended that further research obtain clients’ perceptions of ‘doing dying well’ in specialist palliative care, capturing their perceptions of occupational engagement and whether the components of ‘doing dying well’ developed in this study are fully representative of their needs. Further qualitative research exploring occupational therapists’ perceptions and educational experiences surrounding death and dying could also inform practice.

## Conclusion

The facilitation of ‘doing dying well’ in collaboration with clients and their families and caregivers adds a new dimension to the understanding of occupational therapy in specialist palliative care. The core elements of ‘doing dying well’ were identified from daily experiences of assisting clients to continue engaging in both ‘the little things’, whose significance and meaning may alter or intensify during palliative care, and occupations with special meaning. Involving clients’ families and caregivers in the therapeutic process through education and support can promote ongoing client engagement in meaningful occupations during their palliative care, a capacity which has also been highlighted in previous research (Eva and Morgan, 2018; Hammill et al., 2019; Pizzi, 2015). ‘Doing dying well’ highlights occupation as the core of therapeutic practice and affirms humans as inherently occupational beings throughout life until death.

**Key findings**

- Clients facing death prioritise ‘doing living well’ through meaningful, everyday occupations.
- Client-centred, holistic occupational therapy fosters dignity, identity and empowerment until death.

**What the study has added**

This study reframes palliative occupational therapy by highlighting how client-centred, occupation-focused practices enable individuals to maintain identity, agency and meaning, enriching the concept of ‘doing dying well’.
